# Welding Techniques for High Entropy Alloys: Processes, Properties, Characterization, and Challenges

**DOI:** 10.3390/ma15062273

**Published:** 2022-03-19

**Authors:** Merbin John, Orlando Diaz, Andres Esparza, Aaron Fliegler, Derek Ocenosak, Carson Van Dorn, Udaya Bhat K., Pradeep L. Menezes

**Affiliations:** 1 Department of Mechanical Engineering, University of Nevada, Reno, NV 89557, USA; merbinjohn@nevada.unr.edu (M.J.); odiaz@nevada.unr.edu (O.D.); andyesparza57@gmail.com (A.E.); ajfliegler@gmail.com (A.F.); derek.ocenosak@nevada.unr.edu (D.O.); cgvandorn@gmail.com (C.V.D.); 2 Department of Metallurgical and Materials Engineering, National Institute of Technology, Surathkal 575025, India; udayabhatk@gmail.com

**Keywords:** welding, fusion welding, solid-state welding, high entropy alloys, microstructure, mechanical properties

## Abstract

High entropy alloys (HEAs) are the outstanding innovations in materials science and engineering in the early 21st century. HEAs consist of multiple elements with equiatomic or near equiatomic compositions, which exhibit superior mechanical properties, such as wear resistance, fatigue resistance, and corrosion resistance. HEAs are primarily used in structural and functional applications; hence, appropriate welding processes are essential to enhancing the performances and service lives of HEA components. Herein, a comprehensive overview of current state-of-art-of welding techniques for HEAs is elucidated. More specifically, the article discusses the fusion-based welding techniques, such as gas tungsten arc welding (GTAW) and laser beam welding (LBW), and solid-state welding techniques, such as friction stir welding (FSW) and explosive welding (EB), for a broad category of HEAs. In addition, the microstructural features and mechanical properties of HEAs welded using different techniques were explained for a broad spectrum of HEAs. Finally, this review discusses potential challenges in the welding of HEAs.

## 1. Introduction

For decades, scholars working in materials science and engineering have used pure metals for diverse applications. However, to cater to the needs and demands of applications that require superior mechanical properties, they have introduced the concept of alloying. The alloys can provide remarkable improvements in mechanical properties. Alloying is considered the global cure to many problems associated with strength, ductility, and other properties of pure metals [1]. The idea of using a single principal element with other secondary elements was widely adopted to obtain superior mechanical properties based on needs and demands. This way of alloying techniques has been used for centuries, and scholars have followed these practices. To enhance the properties of these alloys, scholars introduced various surface modification techniques based on severe plastic deformation (SPD) and changes in material manufacturing routes based on property and performance correlations [2,3,4,5]. However, this traditional principal element approach has limitations based on the quantity and number of alloying elements. In addition, these new manufacturing routes and surface modification techniques have limited influences on mechanical properties, and they are expensive. Therefore, scholars searched for a new alloying concept that uses more than one principal element led to the development of high entropy alloys (HEAs) [6,7,8,9].

The foundational work of Yeh et al. [10] and Cantor et al. [11] introduced the novel idea of using five or more principal elements in concentrations ranging from 5 to 35 at.%, and they have named these alloys as HEAs. These ‘HEAs possess four different core effects, such as high entropy effect, sluggish diffusion, severe lattice distortion, and cock tail effect [12,13,14,15]. HEA opened a new path for exploring new metallic materials with remarkable mechanical properties [16,17,18]. The introduction of more principal elements can lead to several phases and intermetallic compounds based on the knowledge of phase diagrams. This concept was highly contradicting to the alloying using a single principal element. However, their hypothesis was that alloying using multiple principal elements in equiatomic or equimolar compositions can cause the configurational entropy of mixing to a value greater than the enthalpy of compound formation. The high entropy effect stabilizes the solid solution phases, such as body-centered cubic (BCC) and face-centered cubic (FCC) phases, and hinders the formation of harmful intermetallics or complicated phases [19]. In addition, HEAs possesses superior mechanical properties, such as high strength and ductility [20,21], superior hardness [22,23,24,25], improved wear resistance [26,27,28,29,30], excellent oxidation and corrosion resistance [31,32,33,34,35,36], high fracture toughness [37,38], excellent high-temperature properties [8,39,40,41], and improved fatigue properties [42,43,44,45,46,47].

Welding is a complex joining technique that can produce permanent joints. Exploring different welding techniques and understanding the welding metallurgy of HEAs in each welding process is of utmost importance in different applications. Proper welding processes with optimized parameters can play a pivotal role in attaining weldability and joint integrity. Weldability and integrity are two important factors considered during welding for a particular application. The welding of similar and dissimilar HEAs expands their applications and makes them a versatile category of materials for structural and functional applications [48,49,50]. Each welding technique has its characteristic properties, and they differ in mechanism, welding parameters, joint preparation, filler wires, inert gas, and substrate thickness [51,52]. The performance of the weld joint can be analyzed by conducting mechanical and metallurgical characterization. HEAs is a hot research topic that sprung up during the past two decades. Understanding the weldability of HEAs is important, as researchers can prevent the potential issues associated with weldability by altering the chemical composition of the substrate. Scholars demonstrated that HEAs could be adopted for diverse applications in industries such as marine, automotive, aerospace, nuclear, and chemical ones [53,54,55,56,57,58]. Several welding techniques have been applied to HEAs. Welding techniques are classified into fusion welding and solid-state welding techniques. The fusion welding techniques are further classified into gas metal arc welding (GMAW), gas tungsten arc welding (GTAW), laser beam welding (LBW), resistance spot welding (RSW), electron beam welding (EBW), and so on. The solid-state welding techniques include friction stir welding (FSW), explosive welding (EB), diffusion bonding (DB), brazing, soldering, and so on. However, this manuscript specifically discusses GTAW, LBW, FSW, and EB of HEAs [48].

This review paper comprehensively discusses the prominently used welding techniques for HEAs. Section 2 describes the historical perspective on HEA development and the weldability of HEAs. Next, the fusion-based welding techniques are explored in Section 3. Furthermore, solid-state welding techniques of HEAs are described in Section 4. Then, Section 5 summarizes the potential challenges related to welding of HEAs. A detailed description of mechanical properties, microstructural changes, and characteristics are summarized for each welding technique. Finally, potential challenges associated with the welding of HEAs are explained.

## 2. HEAs’ Development and Weldability: A Historical Perspective

In traditional physical metallurgy, a pure metal with attractive properties is used as a foundation. Then, alloying concepts were introduced to improve pure metals’ overall physical, chemical, and mechanical properties. Alloying has been an incredibly effective approach in creating stronger and more reliable materials for many years. Still, researchers reached a natural limit of this type of approach as time passed. Meeting this limit has pushed for more research into HEAs, a vast and uninvestigated space in materials science and engineering. Traditional physical metallurgy principles and new methods have been applied to HEAs, which have produced new materials with impressive properties. The new results have shown that traditional materials science concepts do not adequately explain the newly observed behaviors of HEAs fueling new models for complex and concentrated alloys. As a result, the whole field has advanced dynamically and promptly in materials science and engineering [59].

The first relevant results on HEAs were published in 2004. The new concepts of HEAs opened a pathway for research in alloy composition and the potential applications of these materials [7]. The concept of HEAs was first demonstrated by Yeh et al. [10] and Cantor et al. [11] in 2004. They defined HEAs as combinations of five or more elements with atomic percentages higher than 5% and less than 35% [60]. This definition inherently includes all alloys with small atomic percentages of elemental additives because it only requires the primary elements in the alloy to be within that 5–35 at.%.

Even though the original definition of HEAs was five or more elements with atomic percentages between 5% and 35%, the field now includes materials with as few as three principal elements. Therefore, the maximum element concentration may be higher than 35 percent. HEAs can also be called compositionally complex alloys, multi-component alloys, or multi-principal element alloys, and these names are interchangeably used [12,61,62,63]. However, the most widely used name is HEAs. The first HEA developed with good tensile properties was CrMnFeCoNi, which has become the main benchmark for HEAs. It has formed the basis for most of the current scientific understanding of HEAs’ mechanical behavior. These alloys have been observed to have superior properties compared to normal non-HEA-type alloys. The superior properties include good thermal stability, high hardness and strength, excellent wear resistance, electrical properties, magnetic properties, and high corrosion resistance [14]. The need for more advanced alloys to better satisfy the needs of newer technologies is forcing materials science and engineering professionals in the direction of HEAs and multi-principal element alloys (MPEAs). Finding a HEA that will produce superior properties compared to traditional alloys is essential to advancing the capabilities of material scientists and engineers.

**Figure 1:**
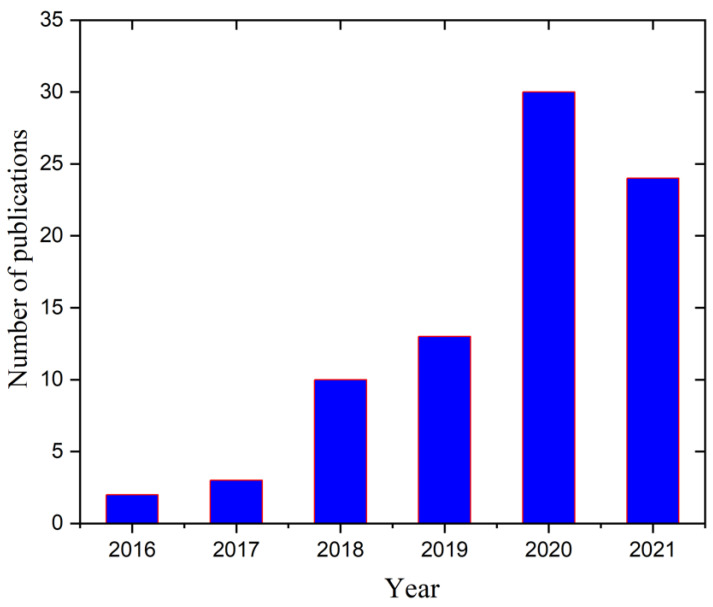
The number of published articles on “Welding of HEAs, 2016 to 2021 from the Web of Science.

The weldability of HEAs is a predominant factor in diverse industries. Researchers faced many issues in the early stages using different welding techniques for HEAs. The welding of HEA-type materials is an area with low levels of research and development. The first research article related to using an HEA as a filler material for welding was published in 2016 [64]. In this work, the authors designed a combination of FeCoNiCrCu HEA and Ti foil as potential filler wire for joining a ceramic and a superalloy using brazing. Following that initial publication on welding techniques for HEAs, an article was published that described the weldability of CrMnFeCoNi for the first time using an EBW [65]. The research and innovations related to the welding of HEAs have continued to increase with the rapid development of HEAs. Figure 1 shows the number of published scientific articles on welding for HEAs from 2016 to 2021, demonstrating the exponential growth of research related to welding of HEAs.

Brazing, laser welding, and FSW make up a large portion of the total number of reviewed and published research papers [66]. The research related to welding of HEA-related materials has continued to increase with the rapid development of HEAs.

**Figure 2:**
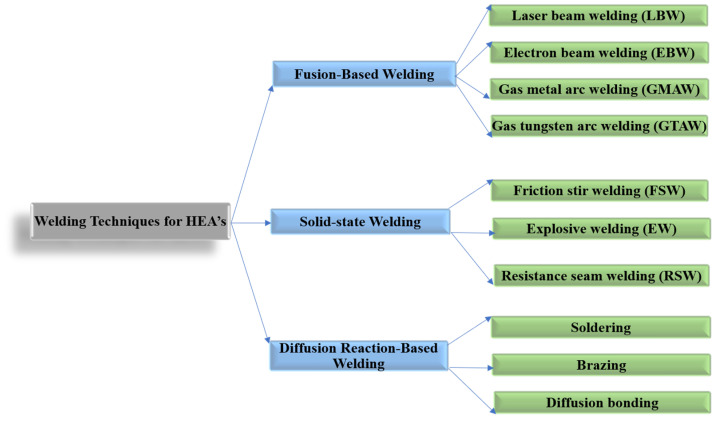
Welding techniques for HEAs.

Over time, three main welding groups have been explored and are shown in Figure 2, where the sub-welding techniques are specified. The welding techniques were elucidated for a broad spectrum of HEAs. Each method has been observed to have positive and negative effects on the properties of the HEAs once the welding was completed. That is why the exploration of multiple techniques of welding is required. Over time, the methods have become the solutions to many problems and have increased the value of HEAs in industrial manufacturing, materials science, and engineering fields.

The major portion of the HEA studies have been focused on understanding the compositions, and the microstructure characteristics and their impacts on actual versus expected properties [67,68,69]. Over time, research on HEAs has become more application-orientated, which requires delivering the predicted properties on a more consistent basis. HEAs have much positive potential to alter the industrial applications of alloys with their superior properties. The need for HEAs has gradually driven many discoveries and advances in many fields of interest.

## 3. Fusion Welding Techniques for HEAs

In the fusion welding method, the materials to be joined are melted with the energy from a high-intensity arc, laser, or electron beam, and then the melt pool is allowed to solidify [70]. Many studies have been conducted on increasing and improving current fusion welding techniques for HEAs. The common techniques studied and reviewed for fusion welding are GTAW and LBW. Each of these welding techniques has its characteristics and applications. These days, more attention is given to the LBW of HEAs than the EBW and GTAW of HEAs [71,72,73]. Parameter optimization in metal arc welding can develop defect-free joints with superior performance at a lower cost than LBW and EBW [74,75,76,77].

The mechanical and microstructural properties are significantly different in various HEAs, because they contain multiple principal elements and are manufactured differently. When fusion welding is performed on HEAs, different phase transformations occur in the weld based on the weld thermal cycle, especially in the fusion zone. Fusion-welded joints can be divided into three regions: fusion zone (FZ), heat-affected zone (HAZ), and unaffected base material (BM) surrounding the HAZ. The HAZ region can be classified into coarse grain heat-affected zones (CGHAZ) and fine grain heat-affected zones (FGHAZ). The fusion zone is generally characterized by columnar grains. In the HAZ region, precipitation of new phases is observed, along with solid-state phase transformations and grain coarsening.

### 3.1. Gas Tungsten Arc Welding

GTAW is a conventional welding process that can produce sound joints with superior weld properties. During GTAW, the base metals to be joined are melted by a non-consumable tungsten electrode. Helium or argon shielding gas protects the weld joints from any atmospheric contamination. GTAW is a welding technique for diverse industrial applications. Scholars have produced various studies revealing similar and dissimilar welding of HEA. Some aspects of those studies are explained briefly.

**Figure 3:**
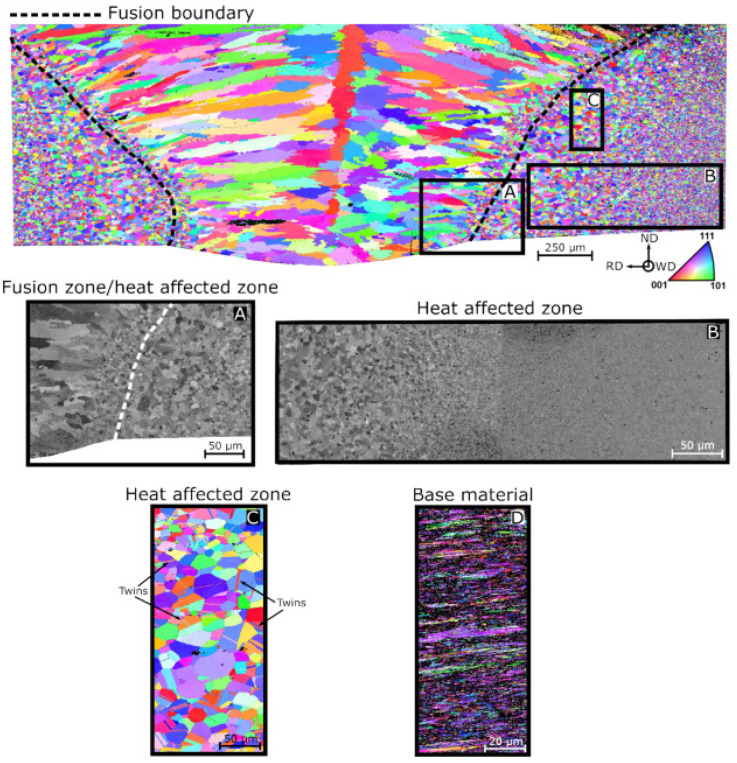
EBSD analysis of the CrMnFeCoNi HEA weld joint: (**A**) the fusion line, (**B**) the HAZ, (**C**) the HAZ containing twins, (**D**) the BM. Reproduced with permission from [78]. Copyright Elsevier, 2020.

Wu et al. [65] welded equiatomic CoCrFeMnNi HEA for structural applications. For this purpose, the authors used GTAW with CoCrFeMnNi and studied the microstructure and mechanical properties of the weld metal and HAZ. They used a voltage of 8.4 V, a current of 75 A, and a welding velocity of 25.4 mm/min. The authors reported the absence of solidification cracks and significant microsegregation, and mentioned the superior weldability of these classes of HEAs. The GTAW joints retained 80% of the ultimate tensile strength (UTS) and 50% of the ductility of the BM. They recommend that weld properties be significantly enhanced by properly controlling the oxygen levels. They summarized that further research is required for identifying the applicability of these classes of HEAs to large-scale industrial applications. Oliveira et al. [78] conducted GTAW on the most widely studied HEA “CrMnFeCoNi” system. The authors optimized the welding parameters, and the following parameters were chosen. A welding current of 60 A, welding voltage of 9.2 V, and welding velocity of 4.2 mm/s were used. A defect-free joint with superior mechanical properties was observed. They reported different microstructural features in the weld joint using electron backscattered diffraction analysis (EBSD). Pancaked-shaped grains with 2 µm thickness were reported for BM. The grain size in the HAZ region was significantly higher compared to the BM. Higher grain size was observed in the fusion zone and HAZ interface. The grain size in the FZ and HAZ interface was 30 µm, whereas in the HAZ and BM interface, it was 5 µm. The grain coarsening in the HAZ was due to the effect of the weld thermal cycle. During welding, the centerline of the FZ experienced the highest peak temperature, and it decreased monotonically towards the base material. They also reported that the peak temperature and permanence time at these peak temperatures both promoted grain growth and were higher and longer than those in the HAZ and BM interface. In addition to that, the solid-state phase transformations depended on the temperature and varied exponentially. The authors also reported the presence of 50 µm grains in the weld center. The growth of these columnar grains is epitaxial, which begins from HAZ and progresses towards the weld center. The EBSD analysis of the weld joint is shown in Figure 3. The authors also reported the presence of annealing twins in the HAZ. However, no such features were observed in the FZ. The weld joint indicated the lowest hardness, about 150 Hv in the FZ. The BM hardness was 350 Hv, which increased to 375 Hv at the BM and HAZ interface. This was attributed to recrystallization, which reduced the grain size due to the weld thermal cycle. A reduction in hardness from BM and HAZ interface to 175 Hv was observed in the interface of HAZ and FZ, which was due to the grain growth in the HAZ.

**Figure 4:**
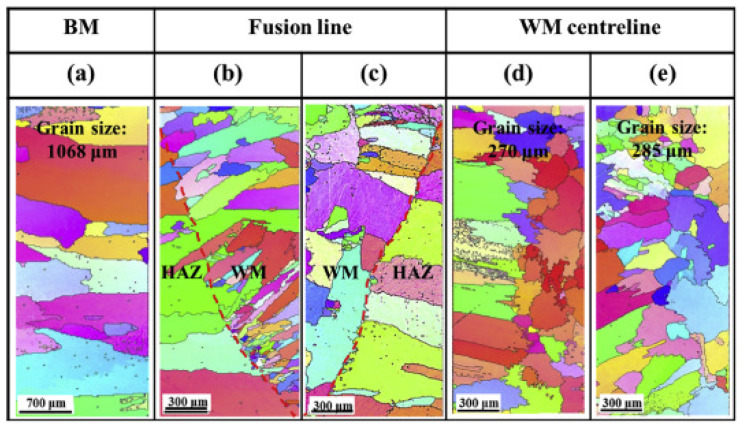
EBSD of (**a**) BM, (**b**) HAZ and WM of HEA fillers, (**c**) HAZ and WM of STS 308 L, (**d**) center of WM of HEA fillers, (**e**) center of WM of STS 308 L. Reproduced with permission from [80]. Copyright Elsevier, 2020.

Furthermore, tensile test results indicated the ductile nature of the weld joint. Reductions in strength and ductility were observed for the weld joint compared to the BM, which were attributed to the changes in microstructure in the FZ and the grain growth phenomenon. Sokkalingam et al. [79] conducted GTAW on Al_0.5_CoCrFeNi plates using a welding velocity of 80 mm/min, a voltage of 12 V, and a current of 40 A. The two plates of HEA material were first heat-treated in a furnace at 1423 K for 24 h and then cooled down in a cooling furnace; then, welding was carried out. The authors observed refined equiaxed grains and elongated columnar grains in the FZ with an average grain size of 8–12 µm. This resulted in reductions of 16.5% in ductility and 6.4% in strength. Nam et al. [80] explored the weldability of CoCrFeMnNi HEA for cryogenic applications. The authors adopted two filler wires in their experiments, stainless steel STS 308 L and HEA. Both filler wires produced superior welds without microcracks, pores, or other defects. In addition, the authors observed FCC crystal structure and the absence of δ-ferrite in WM. The observed grain size for BM was 1 ± 0.2 mm. Figure 4 represents the EBSD analysis of BM, WM, and HAZ regions of HEA welded using STS 308 L and HEA fillers. The columnar grains grew epitaxially from the fusion line to the weld center when HEA filler wires were used. This was due to the same composition of the filler compared to BM. However, incomplete epitaxial growth of columnar grains was reported for STS 308 L filler wires. This was attributed to the different compositions of the 308 L fillers compared to the BM. The transformation of columnar grains to equiaxed grains at the weld center was reported. The dendrite packet size was similar at the weld center irrespective of the filler wire used.

**Figure 5:**
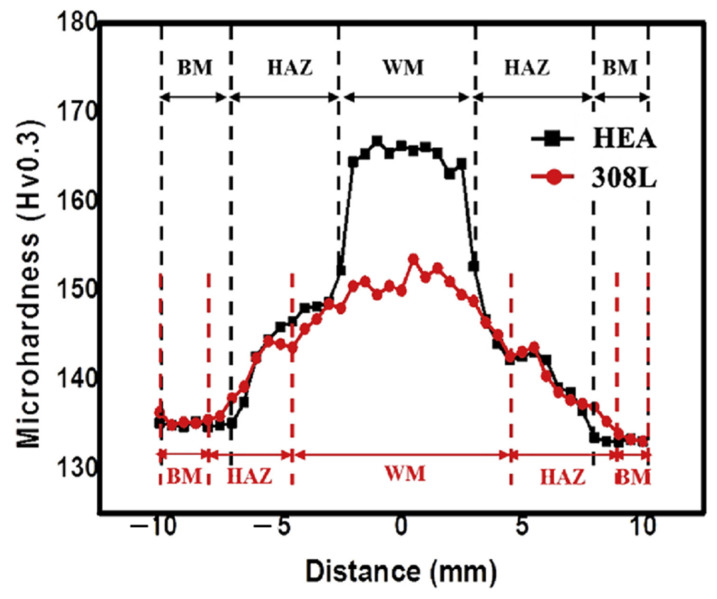
Microhardness distribution across the GTAWed HEA corresponding to STS 308 L and HEA fillers. Reproduced with permission from [80]. Copyright Elsevier, 2020.

The reported average microhardness values in the WM in both filler wire cases are higher than that of the BM. The BM microhardness was 132 ± 1 Hv. The lower hardness of BM was attributed to the larger dendritic size. The WM microhardness corresponding to HEA fillers was 165 ± 1 Hv, and for STS 308 L fillers, 150 ± 1 Hv. The inconsistency in microhardness was because of the differences in grain size, dendrite packet size, and constituent elements in the WM. Figure 5 represents the microhardness distribution in the weld joint.

Sokkalingam et al. [81] demonstrated the requirement of dissimilar welding for aerospace and structural applications. For this purpose, the authors welded HEA with 304 SS using GTAW. The HEA used in this case was Al_0.1_CoCrFeNi. This HEA is most commonly used for power generation applications in chemical and nuclear industries. The welding was carried out with five different welding velocities: 50, 75, 100, 125, and 150 mm/min. The voltage and current chosen for the experiments were 10–11 V and 50 A. The authors reported a weld joint without any macrocracks and pores. The HEA side weldment showed epitaxial growth of grains, beginning from the fusion line and directed towards the weld center, and non-epitaxial growth on the SS 304 side. The dissimilar weld joint showed a yield strength (YS) of 265 MPa and UTS of 590 MPa, significantly higher than those of the BM. The reported YS and UTS of BM are 148 and 327 MPa. Martin et al. [82] studied the weldability of Al_0.5_CrCoCu_0.1_FeNi HEAs using the GTAW technique. The authors reported an absence of solidification cracking and HAZ liquation cracking, demonstrating good weldability. Table 1 shows the mechanical properties and microstructural features of HEA weldments using different fusion welding techniques.

### 3.2. Laser Beam Welding

LBW is an important category of welding among fusion welding techniques. A concentrated laser beam from a laser source interacts with the materials in this process. Due to the high laser intensity, the materials are melted; subsequently, a weld is formed. LBW has advantages, such as high energy density, small HAZ, fast cooling, and high flexibility. The most influential parameters for LBW are frequency, pulse width, and laser intensity [85,86]. In addition, the appropriate selection of process parameters can enhance the weld joint quality. LBW is appropriate for high volume applications and is fundamentally a penetration or keyhole-based welding technique.

Nam et al. [71] explored the possibility of using LBW for cast and rolled HEA for cryogenic applications. The LBW was conducted with an Nd:YAG laser, 3.5 kW, having a beam diameter of 300 µm and a focal length of 304 mm. The authors varied the welding velocity from 6 to 10 m/min. They also optimized the welding parameters to obtain full penetration. The authors observed good weldability and sound joints without macrocracks for all the welding conditions. However, some shrinkage voids were found in the weldment, whose volume fraction was decreased with an increase in welding velocity. The cast HEA welds had full penetration at 6 m/min. However, with an increase in welding velocity, the widths of beads in the top and bottom portions of the welds were decreased, and there was no shrinkage. A similar weld bead and shrinkage voids were observed for rolled HEA, even at 10 m/min.

**Figure 6:**
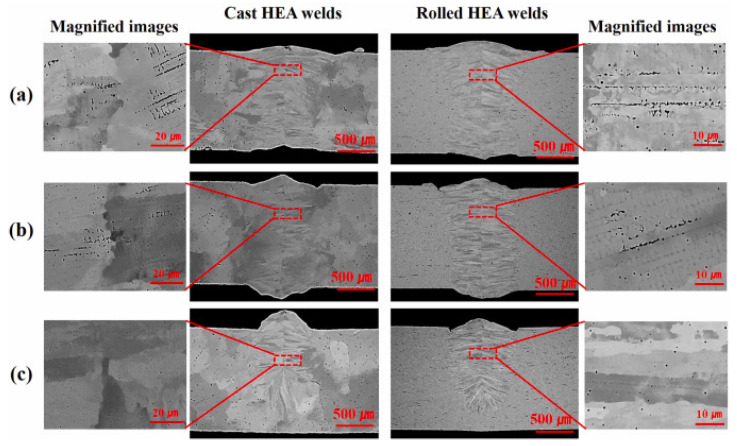
Macrogrpahs of the weld beads for various welding velocities: (**a**) 6 m/min, (**b**) 8 m/min, and (**c**) 10 m/min. Reproduced with permission from [71]. Copyright Elsevier, 2019.

The cast HEA possessed coarse equiaxed grains with a grain size of 1.1 ± 0.2 µm. The HAZ grain size of the cast HEA welds was that of the cast BM. The microstructure near the fusion line was cellular/columnar dendrites, which grew from the fusion line to the weld center, indicating epitaxial growth. The rolled HEA had fine equiaxed grains, and the grain size was approximately 3.3 ± 0.3 µm. The weldment microstructure showed a cellular/columnar dendritic shape similar to the cast HEA weld, which grew from the fusion line, demonstrating epitaxial growth. Improved mechanical properties with a rise in welding velocity were attributed to the reduction in shrinkage voids, reduction in primary arm spacing, and dendrite packet size. There was less hardness in the cast HEA weld compared to the BM. This was attributed to the size of the columnar dendrite in the WM. However, the hardness difference between the WM and BM for rolled HEA was insignificant because of the similar dendritic spacing. Both cast and rolled HEA showed similar tensile properties. Figure 6 represents the macrographs of weld joints of cast and rolled HEAs.

**Figure 7:**
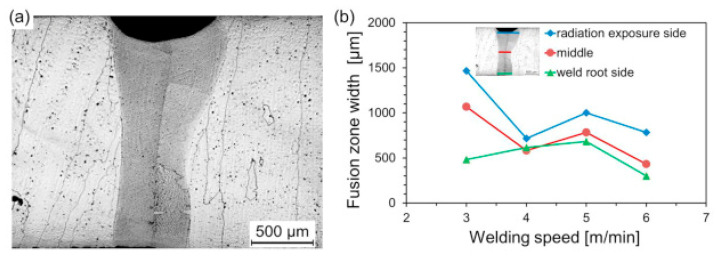
(**a**) Macrograph of weldment corresponding to 5 mm/min, (**b**) variation of fusion zone width with welding speed. Reproduced with permission from [73]. Copyright Elsevier, 2019.

A study conducted by Kashaev et al. [73] performed LBW of CoCrFeNiMn manufactured using a high-temperature synthesis process. The authors adopted a laser power of 2 kW with 300 mm focal length, welding velocity varied between 3 and 6 mm/min, and the HEA plates were kept in butt-joint configuration. The BM had a dendritic structure. TEM studies indicated the presence of rectangular-shaped second phase particles, and they were confirmed to be M_23_C_6_ carbides with selected area diffraction patterns (SAED). A micrograph of the butt joints corresponding to a welding velocity of 5 mm/min is shown in Figure 7a. The fusion line along with the fusion zone width at three different locations in the weld joint corresponding to different welding velocities are represented in Figure 7b. At all welding velocities, no cracks or porosity were reported. The observed weld seam width was ∼570 μm. The authors reported that welding velocities more than 6 mm/min caused partial penetration, and welding velocities lower than 3 mm/min led to a wider weld seam. Welding velocities between 4 and 5 mm/min can provide a cylindrical-shaped weld with superior tensile properties. The authors recommend a weld joint with 5 mm/min welding velocity as optimal based on the weld shape.

Furthermore, after welding, the fusion zone microhardness increased to 208 ± 6 Hv from 153 ± 3 Hv. The higher microhardness in the fusion zone was attributed to the formation of nanoscale precipitates of B2 particles in the weld.

**Figure 8:**
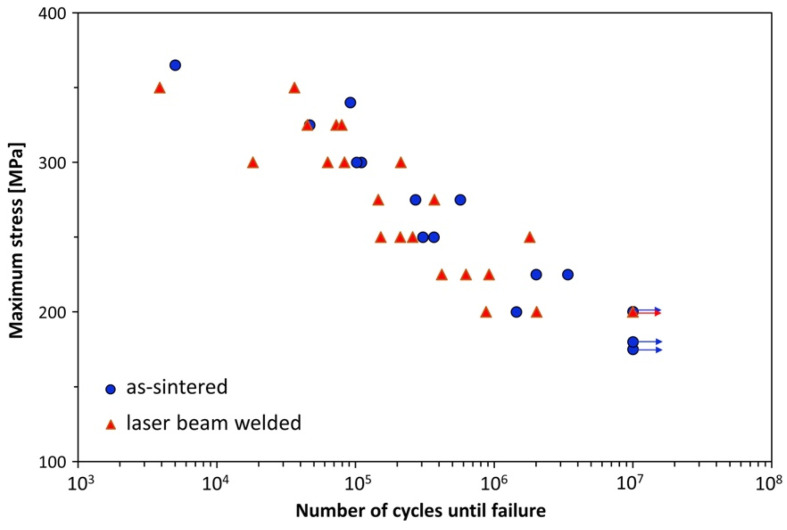
Fatigue test results of LBWed CoCrFeNiMn HEA and BM. Reproduced with permission from [72]. Copyright Elsevier, 2019.

Jo et al. [83] studied LBW on CrMnFeCoNi HEA and reported the microstructural variations and mechanical properties in the WM and HAZ. The reported UTS and ductility of as-weld CrMnFeCoNi were comparable to those of the BM. The fusion zone has a dendritic microstructure, and energy dispersive spectroscopy (EDS) analysis showed higher Fe and Mn content in the interdendritic region. However, they did not observe any intermetallics or other phases in the fusion zone. The hardness of LBW specimens was higher than that of the BM due to the fine dendritic arm spacing. Kashaev et al. [72] studied the fatigue behavior of LBWed CoCrFeNiMn HEA. In their experiments, the authors used a laser power of 2.5 kW and a 5 mm/min welding speed, and the plates were kept in butt-joint configuration. The base material microstructure consists of coarse, elongated, and irregular-shaped grains with a grain size of 250–500 µm. The crystal structure of this HEA was FCC. They also identified manganese sulfide inclusions whose size ranged from 3 to 5 µm both in the fusion zone and in the BM. LBW led to the precipitation of M_7_C_3_-type carbides in the matrix. The reported grain size after LBW was 100–300 µm. Figure 8 represents the fatigue behavior of the BM and LBW specimens. The authors observed no significant difference in the fatigue behavior of BM and LBW specimens. The endurance limit reported for both BM and LBWed specimens was 200 MPa. This demonstrates the superior quality of weld joints compared to the BM. Normally weld joints are weak and are easily susceptible to fatigue failure. Here, the presence of hard fusion zone and intrinsic hardening due to carbide precipitation accounted for similar fatigue properties in LBWed HEA compared to BM.

The authors also reported similar YS and UTS for welded HEAs and BM. However, the ductility of the weld joints was 2% less than that of the BM. Tensile tested weld joint failure occurred far from the weld. Sokkalingam et al. [87] conducted LBW of Al_0.5_CoCrFeNi HEA and studied the corrosion behavior. Potentiodynamic polarization (PDP) tests were conducted to study the corrosion resistance. The corrosion current density, corrosion potential, was identified from PDP. A laser power of 1.5 kW and a 600 mm/min welding velocity were used. The BM microstructure contained equiaxed grains. The width of WM on the top was 2.1 mm, and at the bottom was 1.2 mm. The microstructure of the weld center was columnar dendrites. The authors observed higher corrosion current density in the WM compared to BM. Additionally, the WM corrosion potential was higher (nobler) than that of the BM. Due to this reason, the WM acted as a cathode, and BM acted as an anode. Thus, the weldment (WM + BM) showed lower corrosion current density (less electron flow), indicating enhanced corrosion resistance.

**Figure 9:**
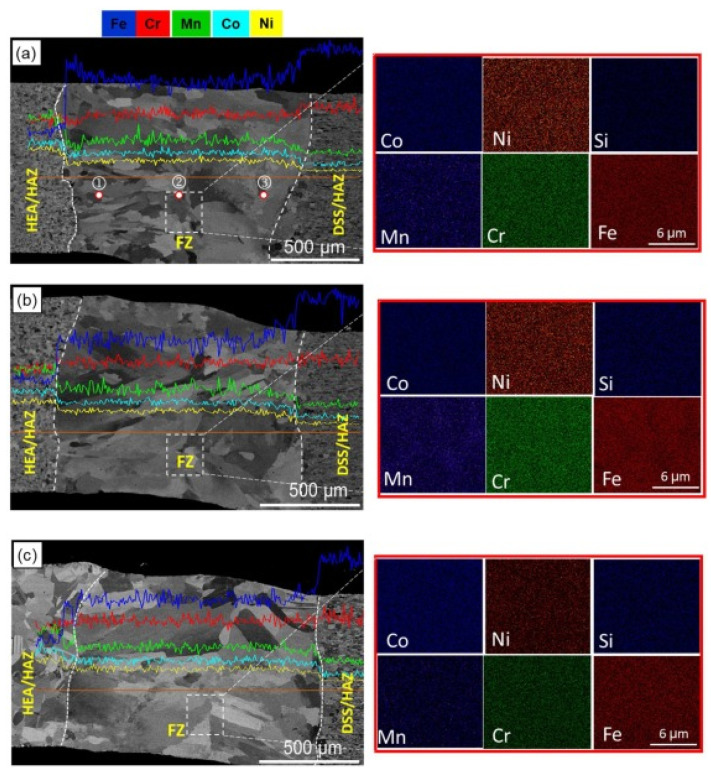
SEM/EDS analysis of (**a**) as-weld, and PWHTed joint (**b**) 800 °C (**c**) 1000 °C. Reproduced with permission from [88]. Copyright Elsevier, 2021.

Post weld heat treatment (PWHT) is always performed to enhance the efficiency of the weld joint. By adopting PWHT, the residual tensile stress (RTS) on the weld joint that arises during welding can be eliminated to a great extent. However, the presence of RTS can lead to inferior corrosion, wear, and fatigue properties. This limits the service lives of weld joints for structural applications. Hence, scholars always recommend using PWHT for enhanced joint efficiency, joint integrity, and longevity of the weld joint. Nam et al. [84] conducted PWHT on LBWed cold-rolled CoCrFeMnNi. They used a welding velocity of 5 to 10 mm/min with a laser power of 3.5 kW. A rise in welding velocity increased the widths of the upper and lower weld beads, and subsequently, partial penetration was observed at 10 mm/min. PWHT was conducted at 800, 900, and 1000 °C. After PWHT, the weld showed comparable tensile properties and hardness to those of the base material. However, they reported that tensile specimens after PWHT failed at the weld center due to the larger grain size. Adomako et al. [88] revealed requirements for dissimilar metal welding for different structural applications in nuclear and aerospace industries. To demonstrate this, the authors performed LBW of CoCrFeMnNi with duplex stainless steel (DSS). After welding, the joints underwent PWHT at 800 and 1000 °C. The authors observed good weldability, and there were no cracks or porosity in the welds. This reveals that HEA can be welded to DSS. The EDS analysis of the weld and PWHTed weld joints corresponding to 800 and 1000 °C is shown in Figure 9.

**Figure 10:**
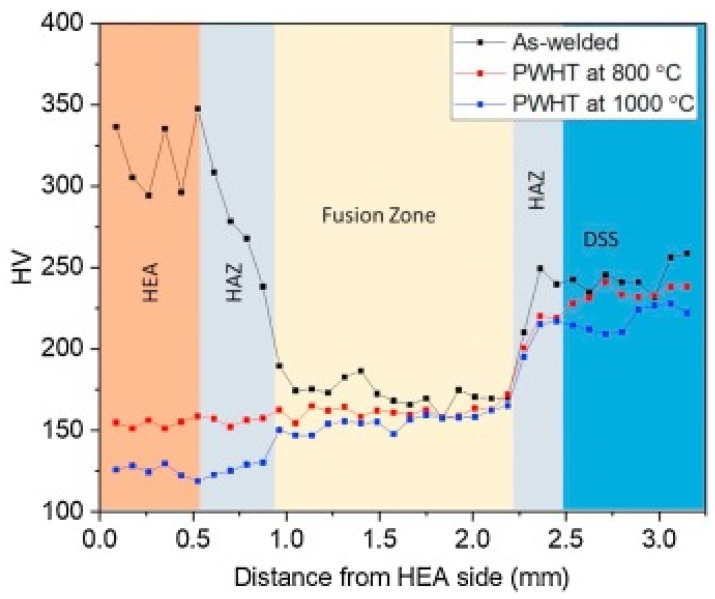
Variation in microhardness of the as -weld is shown in the black line, PWHT at 800 °C indicated in red line or PWHT at 1000 °C is shown in the blue line. Reproduced with permission from [88]. Copyright Elsevier, 2021.

EDS analysis of the weld revealed good mixing of elements across the WM and HAZ. There was no intermetallics formation or microsegregation in the fusion zone, which is expected in dissimilar metal welding. This was attributed to the high solubility at high temperatures in dissimilar metals combinations. PWHT did not influence elemental distribution in the WM and HAZ. Similarly, no precipitates or intermetallics were observed after PWHT. Figure 10 represents the hardness of HEA and HAZ after PWHT. The HEA had a hardness of 320 Hv, which was reduced to 230 Hv in the HAZ. This reduction was due to annealing occurring due to the weld thermal cycle, which eventually caused recrystallization and grain growth. The hardness dropped to 180 Hv in the FZ. The hardness corresponding to DSS in the FZ side was 168 Hv. This mismatch was attributed to the alignment of smaller coarse grains near the HEA side than larger coarse grains on the DSS side. With the rise in PWHT temperature further, decreased WM hardness in both HEA and DSS was observed. This reduction in hardness was more pronounced on the HEA side in both the WM and the HAZ. This was due to the larger grain size on the HEA side than the DSS side after PWHT.

## 4. Solid-State Welding

The fusion-based welding techniques, such as GTAW, LBW, and EBW, work by melting and solidification of WM. There are some potential issues associated with fusion-based welding techniques, such as HAZ softening and a high width for HAZ. This can lead to failure during a dynamic working environment. However, these issues can be minimized significantly by carefully selecting process parameters. On the other hand, some of these issues can be potentially eliminated with solid-state joining techniques.

### 4.1. Friction Stir Welding

**Figure 11:**
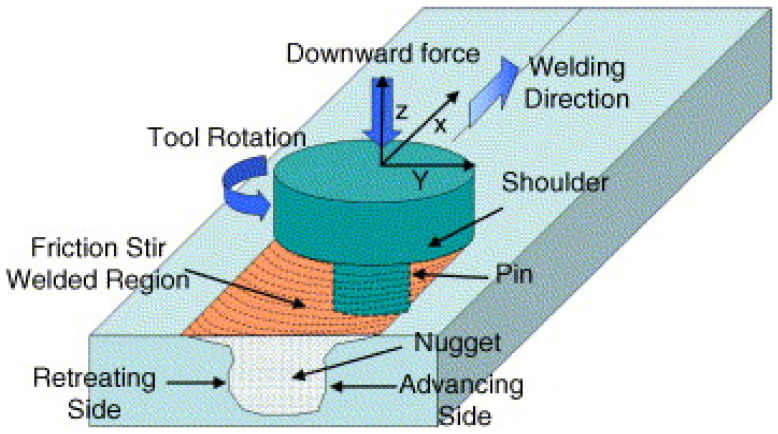
Schematic of FSW process. Reproduced with permission from [95]. Copyright Elsevier, 2005.

FSW is a newly developed solid-state joining technique that possesses superior properties because of the lower heat input during the process compared to fusion welding. The FSW processes have advantages over the traditional welding methods. The process does not involve the melting of metal, which reduces the chances of defects forming during the solidification stage and results in a highly repeatable and pure process. Scholars revealed that FSW could successfully join a wide variety of similar and dissimilar materials [89,90,91,92,93]. In this process, a rotating non-consumable tool with a shoulder containing a threaded pin (probe) at the bottom is used to join the substrate. The shoulder is in proper contact with the top surface of the substrate. During welding, the material is transferred from the advancing side of the tool to the trailing side and becomes forged into a joint. The rotary speed of the tool is such that it will generate heat on the substrate to be joined, and it fills the cavities because of the plastic deformation [94]. The schematic of FSW is shown in Figure 11.

**Figure 12:**
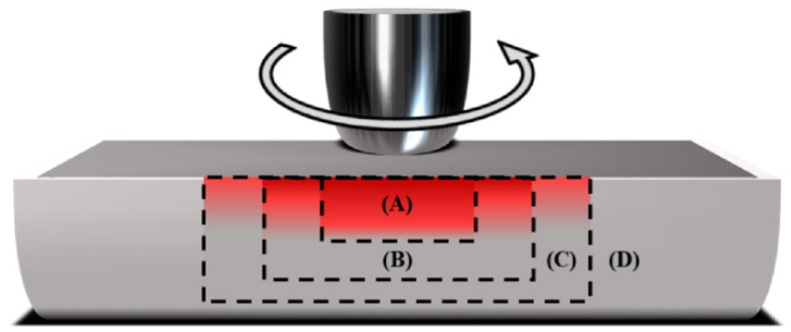
Various zones in the FSW process. Zone A is SZ, zone B is TMAZ, zone C is HAZ and D is BM. Reprinted with permission from reference [93]. Copyright MDPI, 2021.

Generally, the weld zone can be categorized into four zones during the FSW process: stir zone (SZ), thermomechanically affected zone (TMAZ), HAZ, and BM. The TMAZ is in between the SZ and the HAZ. The microstructure in the weld joint and HAZ is entirely different from the fusion welding microstructure. In the TMAZ, the original microstructure is retained; however, it is in a deformed state. Severe deformation is experienced in the SZ [95,96,97]. The highest peak temperature occurs in the SZ, and it has a basin-like shape that is not symmetric about the weld centerline. Figure 12 represents the various zones FSW process.

Zhu et al. [98] conducted FSW on CoCrFeNiAl_0.3_, and they observed a sound weld without any defects. The authors used two welding speeds of 30 and 50 mm/min in their experiments. The tool was rotating at 400 rpm, which had a shoulder diameter of 12 mm, a probe diameter of 4 mm, and a probe length of 1.8 mm. Throughout the process, a constant load of 1500 kg was applied. The authors attributed grain refinement due to recrystallization during FSW as the primary reason for improved microhardness in the SZ. They also observed that welding speed does not influence hardness. In TMAZ, a mixed microstructure comprised of coarse and fine grains was reported because of the partial recrystallization. The authors summarized that the FSW of HEAs could be used for potential future applications.

Park et al. [99] conducted FSW on a cast, rolled Co_0.2_Cr_0.2_Fe_0.2_Mn_0.2_Ni_0.2_ HEAs, and demonstrated the effect of initial grain size on weldability during FSW. The cast HEA had an average grain size of 308 μm, and the rolled HEA had an average grain size of 2.8 μm. The shoulder and probe dimensions were 4.5 and 2.5 mm, and the probe length was 1.3 mm. The rotary speed of the tool was 80 rpm, and welding was carried out at 100 mm/min. The tool used in this experiment was WC-Co. The authors observed good weldability in rolled HEA compared to cast HEA. There were no macro defects in the rolled HEA weld. However, the cast HEA weld contained tunnel defects because of the insufficient stirring during FSW. The primary reasons for tunnel defects were low plastic flow and lower heat input. The authors also calculated the unbounded ratio, defined as the ratio of the total difference in the area in the SZ to the unbounded region due to tunnel defects. After FSW, the grain size of the cast HEA weld was 1.8 μm, whereas that of the rolled HEA was 1.4 μm.

**Figure 13:**
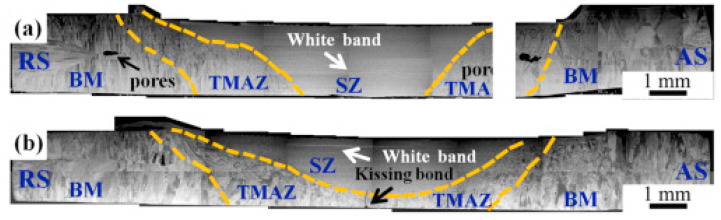
Macrostructure of the weld joint after welding at (**a**) 30 mm/min, (**b**) 50 mm/min Reproduced with permission from [100], Copyright Elsevier, 2018.

Zhu et al. [100] conducted FSW on a highly ductile HEA, with ductility of 70%. The authors used 30 and 50 mm/min welding velocities. The rotary speed of the tool was 400 rpm. A load of 1500 kg with a shoulder diameter of 12 mm, probe diameter of 4 mm, and probe length of 1.8 mm was employed. The authors revealed that the weld penetration decreased with increased welding velocity. This was due to the reduction in weld heat input, which can be seen from the macrographs presented in Figure 13. Figure 13a,b shows the macrostructures for 30 and 50 mm/min welds, respectively. The authors observed superior weld joint integrity without defects, and the FCC phase was retained in the SZ even after welding. Refined microstructures were reported in the SZ compared to the microstructure in the BM. The authors revealed that strengthening in SZ was due to grain refinement during FSW.

**Figure 14:**
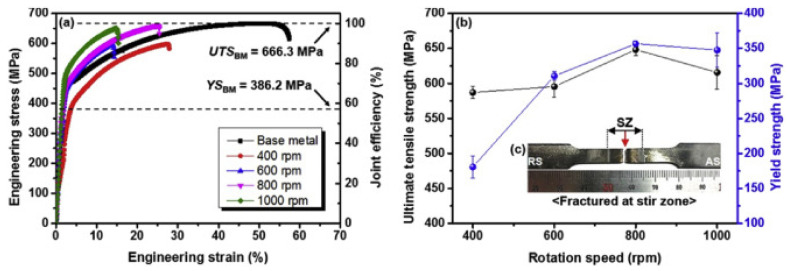
Tensile properties of the FSWed HEA (**a**) stress-strain curve, (**b**) UTS and YS of the joints (**c**) tensile specimen. Reproduced with permission from [101], Copyright Elsevier, 2019.

Park et al. [101] conducted FSW on Co0.2Cr0.2Fe0.2Mn0.2Ni0.2 and studied the effect of rotational tool speed on the weld joint quality. In addition to that, they studied the effects of a tungsten carbide and chromium carbide tool on mechanical properties and correlated these with grain refinement in the SZ. The authors adopted four rotational tool travel speeds, 400, 600, 800, and 1000 rpm, and they maintained a welding velocity of 30 mm/min. The tool was a WC-Co with a shoulder diameter of 4.6 mm, a probe diameter of 2.4 mm, and a probe length of 1.4 mm. The authors reported sound joints without cracks and voids corresponding to rotational tool speeds. High rotational speeds resulted in thinning along the weld line compared to the BM. However, the authors reported an abnormally shaped tornado in SZ after 600 rpm and higher rotation speeds, which appeared from an abundance of carbides entering the material due to tool wear. The carbides in the tornado region were smaller than 0.5 μm, so grain refinement was promoted with the lower rotational speed of the tool. The authors reported that 800 rpm produces superior mechanical properties, despite tool wear. The smallest grain size was achieved at this speed, resulting in the strongest joint. However, when the rotational speed of the tool exceeded 1000 rpm, entrapment of coarse tungsten carbide and chromium carbide particles was observed, and it reduced the grain refinement in the SZ and led to inferior mechanical properties. Figure 14 shows the effects of the rotational speeds of the tool on YS and UTS. From the figure, it is evident that with an increase in rotational speed of the tool, the UTS and YS were increased up to 800 rpm. An increase in travel speed beyond 800 rpm caused a reduction in YS and UTS. The joint efficiency corresponding to 400 rpm was 88%, which increased to 95% for 800 rpm.

Shaysultanov et al. [91] conducted FSW experiments on carbon-doped CoCrFeNiMn HEA. The authors reported that the weld joint possessed properties similar to the BM, attributed to the precipitation of the M_23_C_6_ carbides and grain refinement. Gupta et al. [102] did FSW experiments on the FCC-dominant metastable HEA and revealed superior mechanical properties in the weld region and HAZ. The highest tensile strength was observed in the SZ region, attributed to the refined equiaxed microstructure. Qin et al. [103] studied the influences of FSW welding parameters on mechanical properties and microstructural features of CoCrFeNi HEA. The authors optimized the welding parameters, and they observed a UTS of 627 MPa and ductility of 42% in the optimized condition, which are comparable to those same qualities of the BM. Defects are, of course, still possible with this technique. Contamination, tunneling, and incomplete penetration of the joined metals are examples of such possible defects [50]. However, all these defects can be mitigated by optimizing the welding parameters. Therefore, the potential defects can be overlooked in most applications, as studies show that this affected area of the joined metals experiences a noticeable decrease in grain size and an increase in hardness, predominantly in the stir zone, even with the presence of defects. The improved properties of the weld joint suggest FSW is a very promising welding technique for the future of HEA. As more studies are being published, it is becoming apparent that this welding method is an easily controllable process that produces excellent joints with minimal chances of defects. Table 2 shows the mechanical properties and microstructural features of HEA weldments using different solid-state welding techniques.

### 4.2. Explosive Welding

**Figure 15:**
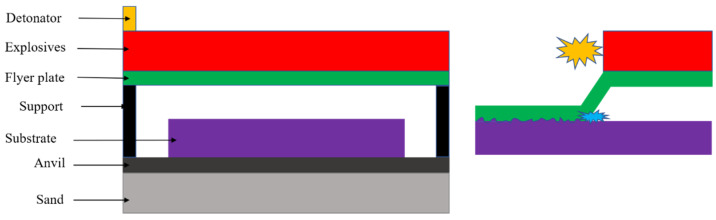
Schematic of the EW technique.

Explosive welding (EW) helps join metals that generally cannot be joined by traditional means. Examples include aluminum and copper, aluminum and steel, aluminum and titanium, titanium alloys, etc. Scholars demonstrated that EW could successfully make dissimilar and similar joints between metals and alloys [105,106,107,108,109]. It is done by forcing two pieces of metal together, similar or dissimilar, at very high velocity and pressure levels using controlled explosives [110]. The impact velocity is high enough that the first atomic layers of each material are joined by diffusion. The deformation zone is subjected to high pressure, leading to a plastic metal jet. The collision essentially eliminates contaminants and defects between the two materials, resulting in a single piece of metal with excellent bonding [48]. A significant amount of heat is generated at the interface; however, the heat transfer to the substrate is minimal because of the short time associated with EW. The strength obtained during EW strongly depends on the microstructure at the interface. The schematic of the EW is represented in Figure 15.

**Figure 16:**
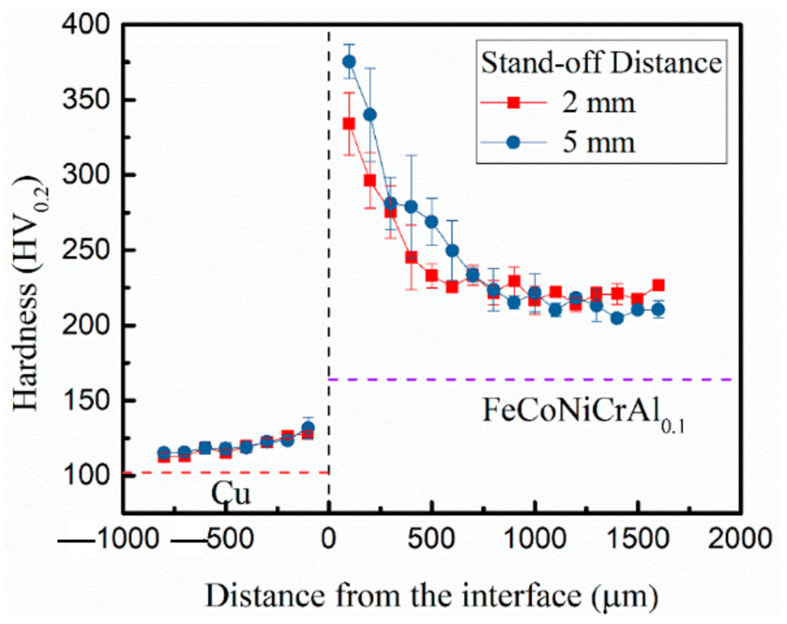
Hardness distribution at the interface of HEA and pure copper. Reproduced with permission from [111]. Copyright MDPI, 2021.

Tian et al. [111] developed a bimetallic composite using EW on FeCoNiCrAl_0.1_ with pure copper used as the flyer plate and studied the interfacial microstructure. The authors observed excellent bonding at the interface, no visible defects, intermetallics in the vortex zone, grain refinement, and phase transformation near the vortex zone due to jetting. They reported lower hardness when the distance from the interface was larger. However, a significant enhancement in hardness was observed near the interface, which was attributed to grain refinement and plastic deformation. The microstructures were elongated at the interface, and due to the excellent thermal conductivity of the copper, the cooling rate at the interface was higher, leading to refined columnar grains. Figure 16 shows the hardness profile corresponding to different stand-off distances of the flyer plate. As the stand-off distance increased, the flyer plate could impact the substrate at higher velocities; thus, the interface had a wavy structure. This situation also consumed more energy, which ultimately enhanced the hardness at the interface.

**Figure 17:**
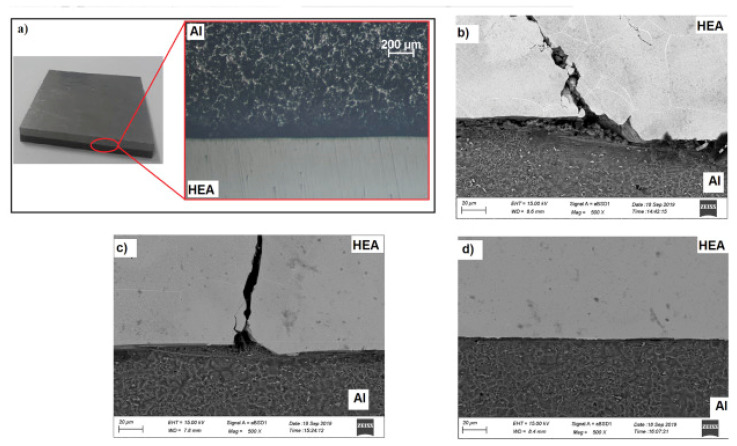
EW of HEA and aluminum: (**a**) optical image, (**b**) stand-off distance: 3 mm, (**c**) stand-off distance 2: mm, (**d**) stand-off distance: 1 mm. Reproduced with permission from [112]. Copyright Elsevier, 2020.

Arab et al. [112] welded HEA and Al-6061 using three different stand-off distances, 1, 2, and 3 mm, so that the flyers struck the substrate with three different velocities. The authors revealed that the aluminum alloy and HEA were properly welded together in all cases. They observed a straight interface without any cracks after using 1 mm stand-off distance. However, they observed cracks after using 2 and 3 mm stand-off distances. The observed crack initiation site was the interface, and from there, it propagated throughout the HEA, and the cracks were due to the formation of intermetallic compounds during the welding process. The authors also reported an improvement in hardness in the aluminum flyer, which was attributed to grain refinement and plastic deformation. Figure 17 shows the SEM of the EW of HEA and aluminum alloy. In the figure, the cracks can be easily seen. As mentioned previously, transverse cracks were observed after using 3 and 2 mm stand-off distances only, as represented in Figure 17a,b. This was attributed to the high impact energy associated with 3 and 2 mm stand-off distances. A shock wave was generated in the interface during EW with the aluminum flyer, causing it to propagate throughout the HEA and reach the free surface of HEA, leading to transverse cracking due to high impact energy.

The observations and findings of these experiments further indicate that EW is a viable technique for use between HEAs and pure metals, which is precisely the advantage of using EW methods for regular alloys and pure alloy metals. However, experiments did not address the actual strength of the resultant joints using this method. Beyond these two experiments, there has not been very much research surrounding the use of EW techniques on HEAs. So far, research has shown that it is a practical way of joining HEAs to pure metals—at least with the HEAs used in the two experiments conducted by Tian et al. [111] and Arab et al. [112]. However, further research must address different HEA compositions and their abilities to join with pure metals and each other through EW.

Although very practical and useful for large-surface-area welds, this method is generally not yet as promising as FSW for HEAs. The primary issue with explosive welding is the creation of cracks in the material that is being welded. The face of the welded materials will be a solid joint; however, the back sides of the two pieces will have cracking due to the impact of the process. However, changes in speed and force can alter this result, suggesting that this method has many potential uses if undesirable results can be mitigated. Even though the discussion on solid solid-state welding of HEA revealed improvements in mechanical properties, more research still needs to be done to advance the emerging field of solid-state welding HEAs and how their excellent properties can be used to make a large impact on the industry.

## 5. Challenges of Welding HEA

This review summarized many experimental papers on HEA welding using fusion-based and solid-state welding techniques. The results showed good weldability and no significant macro defects, pores, or porosity in the weld joints. All the papers discussed in this review considered only butt-joint configuration, the simplest of all joints. However, in the real world, other than the butt joint, there are many other joints, such as lap joints, corner joints, edge joints, and tee joints. Therefore, welding in this configuration may be challenging. Thus, significant research has to be done to identify the influential process parameters for welding in this configuration. It is essential to know the influences of weld heat input on the phase formation, grain size, dendritic pack size, and dendritic arm space to better understand the weld joints’ mechanical properties.

In many cases, a significant drop in hardness from the weld center to the fusion line was observed during dissimilar welding of HEA [113]. This effect is more pronounced during cast HEA welding than rolled HEA. For certain applications, the toughness of the weld joint is more important than the hardness. Under such conditions, the appropriate selection of welding techniques and process parameters is essential. Based on applications and demands, more HEAs are designed every year. Optimizing welding process parameters and appropriate welding techniques is critical to joining these HEAs for various applications [51,66]. Currently, much research related to welding is focused on the most widely used system: CoCrFeNiMn. More in-depth research is required to make various welding techniques for similar and dissimilar welding of other HEAs applicable. Another important challenge during HEAs welding is the appropriate filler wire selection. Based on HEAs’ ultimate tensile strength (UTS) levels, filler wires can be classified into three categories: undermatching filler wire, matching filler wire, and overmatching filler wire. Choosing overmatching filler wire during welding is always recommended to avoid joint failure. Overmatching filler wire has higher alloying content and can provide superior properties in the weldment. However, they are costly because of their higher alloy content. From an economical point of view, it is recommended to choose undermatching filler wire and matching filler wire during welding of HEAs. The field of application intended for the fabricated weld joint needs to be considered during the filler wire selection.

## 6. Conclusions

This review paper provided a comprehensive overview of the welding of HEA. HEA is an outstanding finding in the materials science and engineering domain. The evolution of HEA has helped scholars and scientists to use advanced materials with superior mechanical properties for diverse industrial applications. This review explained the needs for advanced materials and demands for the development of HEAs to meet these requirements. The historical development of HEAs was elaborated to explain HEAs better. Fusion-based welding techniques, such as gas tungsten arc welding (GTAW) and laser beam welding (LBW), were explained for different HEA systems. In addition to that, solid-state welding techniques, such as friction stir welding (FSW) and explosive welding (EB), were summarized for a broad category of HEAs. The microstructural evolution, WM properties, HAZ properties, grain size variation, epitaxial growth, weldability, and weld’s mechanical properties were discussed. Finally, this review explained the potential challenges in the welding of HEAs. This review article can provide deeper insights for the selection of welding techniques for a particular HEA system.

## Figures and Tables

**Table 1:** Mechanical properties and microstructural features of fusion welded HEA.

Material	Welding Technique	Welding Parameter	Observation
CoCrFeMnNi (0.2) [71]	LBW	Nd:YAG laser; Laser power—3.5 kW; Welding velocity—6–10 m/min No shielding gas	Cast HEA weld has similar tensile strength as of BM, whereas rolled HEA has a lesser tensile strength In cast HEAs, tensile fracture occurred near HAZ and BM. In rolled HEA, tensile failure occurred in the WM. In cast HEA side sharp hardness variation is observed of Dendritic arm spacing (DAS) and dendrite packet size were higher for cast HEA compared to rolled HEA
CoCrFeNiMn [72]	LBW	Ytterbium fiber continuous laser; Butt joint configuration; Laser power—2.5 kW; Welding velocity—5 m/min	Improvement in hardness due to the presence of M7C3 type carbides Tensile properties of the welded HEA were identical to BM Tensile failure of the weld joints occurred far from the weld zone Fracture happened near aside of fusion zone
CrMnFeCoNi [78]	GTAW	Current—60 A; Voltage—9.2 V; Welding velocity—4.2 mm/s; Heat input—131.4 J/mm. Direct current straight polarity	Good weldability In the HAZ, due to weld thermal cycle, recovery, recrystallization, and grain growth were observed High tensile strength and reduced fracture strain The strength and ductility of weld joints is less than BM
CrMnFeCoNi [65]	EBW	Current—5 mA; Voltage—125 kV; Welding velocity—9.53 mm/s	Solidification cracks were absent Weld has the same strength level and ductility in comparison with base material at RT and at CT Large number of deformation twins was observed in the fusion zone
CrMnFeCoNi [83]	LBW	Yb:YAG disk laser, Laser power = 3 kW, 3.5 kW, focal length—450 mm, beam diameter—200 μm, welding velocity—9–10 m/min	Compared to BM, laser welded specimens have lower ductility and tensile strength. The microstructure in the FZ is dendritic. Intermetallics were not observed in FZ. High hardness observed for LBWed specimen due to small dendritic arm spacing
Co_0.2_Cr_0.2_Fe_0.2_Mn_0.2_Ni_0.2_ [84]	LBW	Nd:YAG laser, Laser power = 3.5 kW, beam diameter—300 μm, focal length—304 mm, welding velocity—5–10 m/min	No macro defects were observed Hardness and other tensile properties were inferior compared to BM Post welding heat treatment (PWHT) improved the hardness and reduced the size of oxide inclusions PWHT retained FCC structure in the weld and FZ

**Table 2:** Mechanical properties and microstructural features of solid-state welded HEA.

Material	Welding Technique	Welding Parameter	Observation
CoCrFeNiMn [91]	FSW	Shoulder diameter—12.5 mm; Pin length—1.5 m; Tool rotation speed 1000 rpm; Tool travel speed 30 mm/min; Force—11.1 kN	Sound weld without any macro defects were observed BM has a grain size of 9.2 µm, whereas the stir zone (SZ) has a grain size of 4.6 µm The higher volume fraction of M_23_C_6_ carbides were observed in SZ Ultimate tensile strength (UTS) increased by 80 MPa, and yield strength (YS) increased by 200 MPa
CoCrFeNiAl_0.3_ [98]	FSW	Shoulder diameter—12 mm; Pin length—1.8 mm; Probe diameter—4 mm; Tool rotation speed 400 rpm; Tool travel speed 30 mm/min and 50 mm/min; Load—1500 kg	Sound weldwithout any macro defects were observed SZ zone has refined microstructure Themromechancially affected zone showed a mixed microstructure due to partial recrystallization Recommend FSW as a superior joining technique for various engineering applications
CrMnFeCoAl [83]	FSW	Shoulder diameter—12 mm; Pin length—1.85 mm; Probe diameter—4–5.76 mm; Tool rotation speed 600 rpm and 700 rpm; Tool travel speed 150 mm/min	Tensile properties of weld joint are compared to BM Grain size reduced by 114 times compared to the grain size of BM, which is attributed to the high recrystallization tmeperature and short welding duration The hardness of the weld joint was higher than BM
Co_16_Fe_28_Ni_28_Cr_28_ [100]	FSW	Shoulder diameter—12 mm; Pin length—1.8 mm; Probe diameter—4 mm; Tool rotation speed 400 rpm; Tool travel speed 30 mm/min and 50 mm/min; Load—1500 kg	Sound weld without any macro defects was observed SZ has the FCC phase the same as BM Grain refinement was observed in the SZ zone The deformation during FSW is characterized as simple shear
Al_0.3_CoCrCu_0.3_FeNi [104]	FSW	Tool rotation speed 150 rpm; Tool travel speed 60 mm/min	Ductility and strength enhanced after FSW Fine grains were observed in SZ due to partial recrystallization The enhanced properties in HEA is used to low stacking fault energy and high grain growth activation energy Recommended FSW as a special process for enhancing mechanical properties of HEAs
CoCrFeMnNi [99]	FSW	Shoulder diameter—4.5 mm; Pin length—1.3 mm; Probe diameter—2.5 mm; Tool rotation speed 80 rpm; Tool travel speed 100 mm/min	Sound weld without any macro defects was observed After FSW cast side grain size was 1.8 µm and rolled HEA has a grain size of 1.4 µm Rolled HEA has better weldability than cast HEA Cast HEA weld contains high-density high angle grain boundaries and twins

## Data Availability

Data sharing is not applicable to this article.
